# Modern Sociology

**Published:** 1900-08-25

**Authors:** 


### Modern Sociology.

### TO HELP LONE LORN WOMEN.

One of the most valuable of modern societies is that which bears the name of the "Travellers' Aid." It is intended to protect girls and young women from the manifold dangers they may encounter in going from home to strange places. Every day there are large numbers of young women leaving home, usually to take a situation of some kind. Whether as governesses, companions, or domestic servants they are constantly leaving country homes, where they know and are known to everyone around, for London or some other great city. Now, there are other situations than those we have mentioned for which young women—pretty young girls—are wanted. "Things not told in the market lists are bought and sold." Of the advertisements we see in our newspapers for the services of young women a considerable proportion are sham. They are meant to lure girls, not to honest work, but to shame and wantonness. There are very few people who do not know of some case of a girl going to a situation in answer to an advertisement and simply disappearing for ever, or being heard of only to the grief and shame of her friends. We hear occasionally of the kidnapping of girls to the Continent; but the same evil trade goes on here. Foreign girls as well as English ones are lured away, and girls from the country are led to destruction on the very moment of their arrival in large towns. Sometimes the situation they think they are to go to is very different from what they suppose; sometimes they do not know where to go, and are misled by some apparently obliging person at the station or in the street. And always the end is shipwreck.

It is to meet such cases that this society, of which the Lady Frances Balfour is president, has been started. Station visitors attend at the different stations and also at the docks, where many foreigners arrive, and when a girl appears to be bewildered and lonely, they take charge of her, guide her to her destination, if she has a definite one, or, if not, take her to the home and try to find work for her. To persons who travel much it seems strange that a railway or steamboat journey should be so confusing to others. But so it is, and inexperienced travellers make the strangest blunders. To quote one case given in the annual report of the Travellers' Aid Society, a station visitor had gone to meet a girl who was to arrive at Liverpool Street Station, and was much troubled when her charge did not come by the expected train. But the poor girl, who had never made a journey by train before, had come out at the first stopping-place, and was waiting for "the lady who was to meet her" at—Bishop's Stortford. In this case the girl's relations, or someone interested in her, had taken the wise precaution of communicating with the society beforehand. When this is done, the society will make inquiries as to the respectability of the situation to which the girl is going, or of the registry office to which she is applying, or the lodging where she thinks of staying. For a fee of a shilling, in addition to the travelling expenses of the station visitor, which are generally small, a representative of the society will meet "any girl of respectable character, of whatever creed or nation, going to London or any other place where she has not friends." It is surely well worth while for the poorest parents to pay this small sum to make sure that their girl goes safely to respectable work, rather than let her run all the risks that abound in a strange place for an inexperienced young woman.

Besides these cases, which may be regarded as constituting the society's regular work, the station-visitors constantly get all manner of strays on their hands. There are girls who have no friends, and do not know where to go. There are girls whose friends have failed to meet them, and who are, therefore, at a loss what to do next. There are girls who mean to go to an aunt or other relative to whom they have not written, and who has changed her address since the last communication passed between them. And there are foreigners, who bring with them the most mysterious addresses of the friends whom they wish to join. How would the average person direct a girl to "Quns Beldnksgs, Goshl St. Betinaken, gren Rout E., london Englan"? Nothing but experience of such matters, added to native intelligence, would make that out to be "Queen's Buildings, Gossett Street, Bethnal Green Road," though the last part of the address is simple enough.

A very important part of the work is inquiring into the respectability of situations advertised. One case may be given as showing how important it is that such inquiries should be made, and what a good thing it would be if the friends of any young woman would avail themselves of the society's help. "An advertisement was so worded as to give the impression that a young lady was wanted to travel on the Continent with an old lady, who was something of an invalid. An interview was appointed at a London hotel on a Sunday, but the appointment was never kept, for we had to send word to the girl's mother that the one who wanted a companion was neither aged nor an invalid—nor even a woman." Of course, in many cases the inquiries prove that the advertised situation is perfectly respectable, but a case like the one quoted is enough to show how necessary investigation is in every instance. Inquiries have been made by the society in almost every quarter of the globe, though the majority have been in London. But a society whose agency includes, as the year's records show, Berlin, Birmingham, Boulogne, Cambrai, the Cape, Cassel, Cherbourg, Colombes, Cork, Ghent, Lancing, Leicester, Rosbach, Twickenham, Vienna, and Woking is a far-reaching benefit. There are branches of the society in several provincial towns, especially seaports, as well as in Paris, New York, Philadelphia, Melbourne, Adelaide, and other foreign and colonial towns. The society also places placards at railway stations and on board steamers, warning girls not to trust to the representations of unauthorised persons, and telling them where to find the agents of the society. Many girls are directed to the society through these placards, and the railway officials are only too glad to turn over helpless girl travellers to its agents. During the last year 3,031 cases have been dealt with in one way or another. The society would be glad of local workers in every place in the kingdom. It appeals also to railway porters and to stewardesses on ships to ask such questions of young girls who are travelling alone as may put them on the right track. Inquiries may be made of, and subscriptions for this useful work sent to, the secretary, Miss Jessie Gordon, 3, Baker Street, W.

